# Negative impact of reduced exocrine pancreatic function on sarcopenia risk in the asymptomatic general population

**DOI:** 10.3389/fnut.2026.1830761

**Published:** 2026-04-28

**Authors:** Mats L. Wiese, Giancarlo Paradiso, Fabian Frost, Sabrina von Rheinbaben, Martin Bahls, Till Ittermann, Henry Völzke, Markus M. Lerch, Frank Ulrich Weiss, Ali A. Aghdassi

**Affiliations:** 1 Department of Food, Nutrition, Facilities, FH Münster University of Applied Sciences, Münster, Germany; 2 Department of Medicine A, University Medicine Greifswald, Greifswald, Germany; 3 Department of Hematology and Oncology, University Medicine Greifswald, Greifswald, Germany; 4 Department of Internal Medicine B, University Medicine Greifswald, Greifswald, Germany; 5 German Centre for Cardiovascular Research (DZHK), Partner Site North, Greifswald, Germany; 6 Institute for Community Medicine, University of Greifswald, Greifswald, Germany; 7 LMU University Hospital, Ludwig Maximilian Universität München, Munich, Germany

**Keywords:** exocrine pancreatic function, fecal elastase, handgrip strength, muscle mass, sarcopenia

## Abstract

**Background:**

In patients with pancreatic disease, exocrine insufficiency facilitates sarcopenia. Here, we investigated whether impaired exocrine function in the asymptomatic general population is also associated with sarcopenia.

**Methods:**

We pooled data from two independent cohorts of 5,598 participants without history of pancreatic disease enrolled in the Study of Health in Pomerania. The association between impaired exocrine pancreatic function (fecal elastase ≤ 200 μg/g) and sarcopenia was determined by cross-sectional, univariable and multivariable regression analyses. The relation between baseline fecal elastase levels and incident sarcopenia was tested in a subgroup of 1,587 persons with available 5-years follow-up data.

**Results:**

We found a baseline prevalence of 4.1% and 9.1% for sarcopenia and impaired exocrine pancreatic function, respectively. While there was no association in persons with age ≥ 65 years, impaired exocrine pancreatic function was associated with the risk of sarcopenia only in younger subjects (OR [95% CI]: 2.52 [1.40; 4.54]). These crude results remained robust in the multivariable models (OR [95% CI]: 2.41 [1.26; 4.61]). Longitudinal analyses did not show an association between exocrine pancreatic function and incident sarcopenia. However, reduced fecal elastase concentration at baseline was associated with muscle mass decline during follow-up. This relation was age-dependent with a significant inverse association only among younger (β [95% CI]: −0.23 [−0.39; −0.08]) but not older study participants (β [95% CI]: 0.04 [−0.31; 0.39]).

**Conclusion:**

Impaired exocrine pancreatic function is associated with sarcopenia in younger individuals of the general population. Studies are called for testing the effect of pancreatic enzyme replacement therapy in asymptomatic patients with impaired exocrine pancreatic function.

## 1 Introduction

The term “sarcopenia” was originally introduced to describe the age-related decline in muscle mass and function (1). However, the understanding of sarcopenia has evolved since then. Sarcopenia is now considered a skeletal muscle disorder that is not exclusively related to aging but can also be caused by other factors including systemic diseases, physical inactivity, and malnutrition (2, 3). Irrespective of its causes, sarcopenia is linked to a wide range of adverse health outcomes including falls, fractures, and physical disability but also increased morbidity and mortality risks (4–6). Despite extensive efforts, early diagnosis and treatment of sarcopenia remain challenging. In this context, it is crucial to identify individuals who are at risk of being affected by sarcopenia (7).

Especially patients with pancreatitis or pancreatic cancer have an increased risk for sarcopenia (8–11). Pooled sarcopenia prevalences of 39% and 45% have been reported for patients with chronic pancreatis (10) or pancreatic cancer (11), respectively. Among other factors, exocrine pancreatic insufficiency (EPI) resulting in malassimilation, if treated inadequately, is considered a key driver of sarcopenia in these patients. While severe EPI is generally clinically overt presenting in the form of steatorrhea, weight loss or abdominal pain, the mild to moderate form can be asymptomatic for long periods (12, 13). Quantification of fecal elastase is a clinically reliable, noninvasive, and simple test to assess exocrine pancreatic function. The prevalence of EPI in the general population without any known underlying disease is estimated to be between 10% and 20%, with higher rates observed among the elderly (14). However, outside of studies asymptomatic EPI will often remain undiagnosed and untreated. The health implications of subclinical EPI are yet unclear but it is reasonable to assume that compromised exocrine secretion could also accelerate loss of muscle mass and function in the general population. To assess whether impaired exocrine pancreatic function is associated with the risk for sarcopenia in otherwise asymptomatic individuals, we studied the relation between fecal elastase levels with muscle mass and function in a pooled sample of two population-based studies.

## 2 Materials and methods

### 2.1 Study population

**FIGURE 1:**
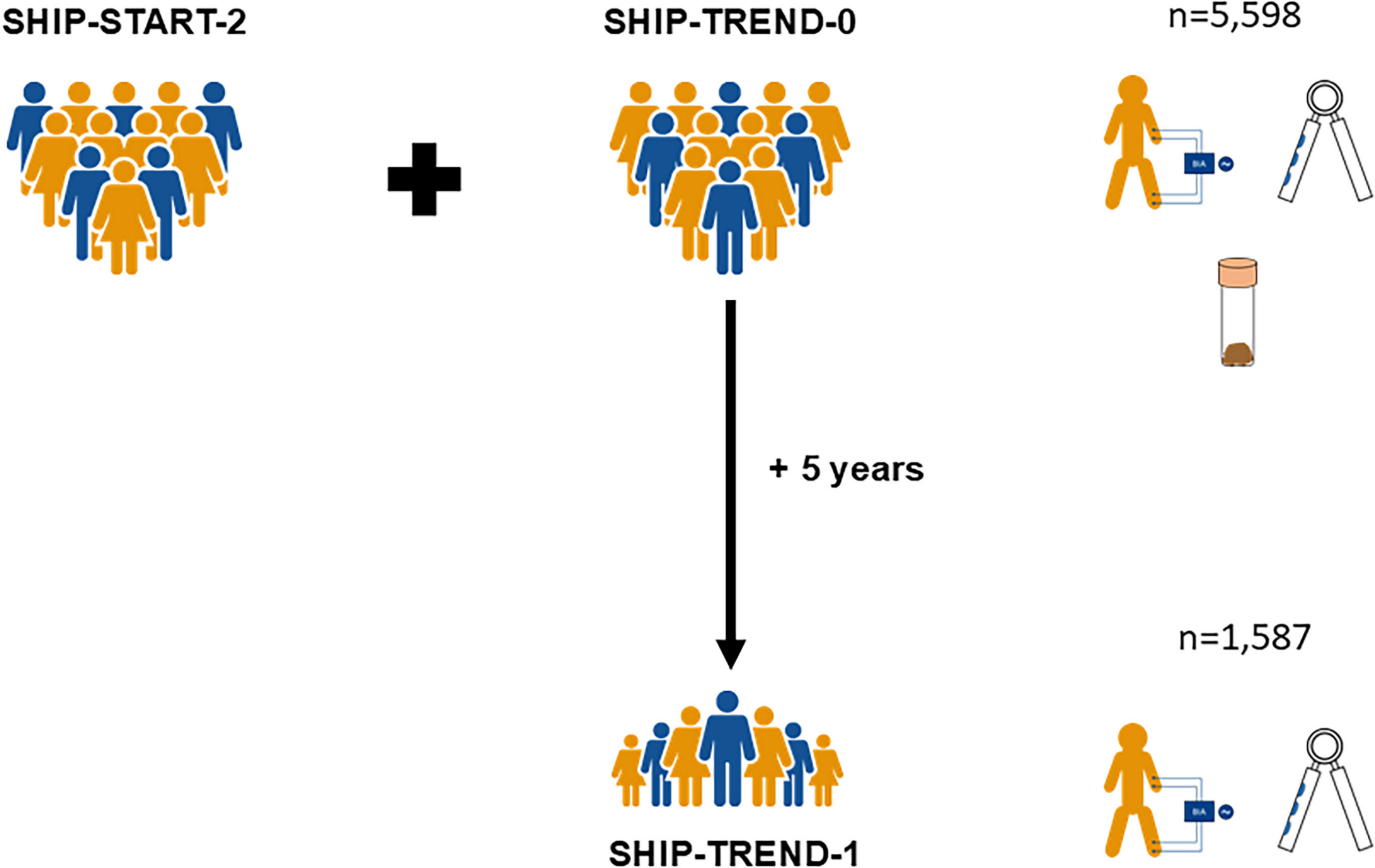
Schematic outline of study design.

For this study, we pooled data from two independent cohorts of participants enrolled in the Study of Health in Pomerania (SHIP). This longitudinally designed population-based project is conducted in Northeast Germany. Its purpose and study design have been previously described in detail (15, 16). SHIP obtained ethical approval by the local institutional review board at University of Greifswald (approval number: BB 39/08). Cross-sectional analyses of the association between pancreatic exocrine function and sarcopenia were performed with pooled data of the cohorts SHIP-START-2 and SHIP-TREND-0, which recruited participants between 2008 and 2012. For the present study, we included all subjects without a self-reported history of pancreatic cancer, acute or chronic pancreatitis as well as complete data on exposure and outcome variables. To substantiate a causal contribution of reduced pancreatic exocrine function to development of sarcopenia, we then performed longitudinal analyses in a subgroup of subjects who also participated in the follow-up investigation SHIP-TREND-1, which took place approximately 5 years after SHIP-TREND-0 (Figure 1).

### 2.2 Diagnosis of sarcopenia

In agreement with current international consensus definition, diagnosis of sarcopenia was based on the finding of concomitant reduced muscle mass and strength (17). We calculated skeletal muscle mass based on bioelectrical impedance analysis (BIA) using the validated prediction equation by Janssen et al. (18). BIA was performed using the multifrequency Nutriguard-M device (Data Input GmbH, Pöcking, Germany) according to the manufacturer’s instructions, as reported in detail before (19). Reduced muscle mass and strength were defined based on sex-specific thresholds. Because reference data for the population of Northeast Germany is not available, we derived cut-offs directly from the population studied. To derive these values, we calculated the 10th percentile of skeletal muscle mass index and handgrip strength for males and females. As recommended by international consensus criteria, we used normative references, i.e., healthy young adults (2). Thus, we excluded all persons 65 years or older and those with any reported malignant or chronic disease or other condition associated with reduced muscle mass or function. The following thresholds were derived: skeletal muscle mass index (males: 9.3 kg/m^2^; females: 6.7 kg/m^2^); handgrip strength (males: 39 kg, females: 23 kg).

Assessment of handgrip strength was performed using the same standardized protocol as described previously (20). Briefly, handgrip strength was measured with a handgrip dynamometer (Smedley’s Dynamometer, Scandidact, Odder, Denmark). Single measurement was taken for the right- and left-hand side, respectively. Participants were standing with their elbow in a 90° flexion and their arm held against the trunk. After 3 s of maximum effort squeezing peak contractile force (kg) was recorded. For the analyses, we used the highest value obtained from both measurements.

### 2.3 Measurement and classification of exocrine pancreatic function

To evaluate exocrine pancreatic function, fecal samples (30–100 mg stool) were collected from the study participants. All samples were evaluated regarding stool consistency to avoid misclassification due to diarrhea. Subsequently, concentrations of fecal elastase were measured in these samples with a polyclonal pancreatic elastase ELISA (BIOSERV Diagnostics GmbH, Germany). The established threshold of 200 μg/g was set to define reduced exocrine pancreatic function, with a concentration of less than 100 μg/g denoting severe impairment.

### 2.4 Statistical analyses

We performed univariable and multivariable logistic regression analyses to test the cross-sectional association between exocrine pancreatic function and prevalence of sarcopenia at baseline. To address confounding, we tested parameters described to be associated with sarcopenia and/or fecal elastase in that regard. These included age, sex, body mass index (BMI), smoking status, alcohol intake, malignancy, chronic kidney disease, dietary intake, diabetes mellitus, and physical activity. In the final multivariable regression models, we included all covariates that met the criteria of potential confounders, i.e., showing an association with exposure, being an independent risk factor but not an intermediary step in a causal pathway between exposure and outcome, based on the analyzed dataset, or existing literature. These were age, sex, BMI, smoking status, diabetes mellitus, and physical inactivity (i.e., less than 1 h of exercise per week). In addition, we tested for a potential interaction between the exposure and these covariates in relation to the sarcopenia risk. We identified a significant interaction for age (impaired exocrine function × age; *p* = 0.004) and therefore stratified the analysis for persons being 65 years or older and those being younger. Age of 65 years was chosen as an internationally established threshold, also denoting statutory retirement age in Germany, and was confirmed as a clinically relevant cut-off point. To investigate the cross-sectional associations of exocrine pancreatic function with muscle mass and strength, we used univariable and multivariable linear regression analyses with fecal elastase levels as an independent variable. In addition, we calculated the ratio of handgrip strength to skeletal muscle mass to obtain a measure of muscle specific strength and analyzed the association of fecal elastase levels with this parameter as well.

For longitudinal analyses, we performed analogous regression analyses with newly onset sarcopenia during the follow-up as the dependent variable for logistic regression models. Likewise, linear regression models were calculated to examine associations between baseline exocrine pancreatic function and changes in muscle mass, strength, or quality.

For all models, potential non-linear associations between exocrine pancreatic function and sarcopenia or muscle parameters were tested by fractional polynomials (21). A non-linear association was assumed when the model with the transformed exposure variable fitted the data significantly better than the model with the untransformed exposure variable.

Statistical procedures were carried out using IBM SPSS Statistics for Windows version 28 (IBM Corp., Armonk, NY, United States) and Stata 18 software (StataCorp LLC, College Station, TX). Descriptive data is reported as mean (±SD) or median (interquartile range, IQR) for normally and non-normally distributed continuous variables, respectively. Categorical data are presented as n (%). Statistical significance was defined as a *p*-value below 0.05.

## 3 Results

### 3.1 Study population

The After exclusion of subjects with missing or incomplete assessment of muscle mass, strength, or fecal elastase levels (*n* = 1,111) and those with a history of pancreatic disease (*n* = 43), a total of 5,598 persons from the SHIP-START-2 (*n* = 2,074) and SHIP-TREND-0 (*n* = 3,524) were included in our analyses. Demographic and participant characteristics stratified by presence or absence of sarcopenia are shown in Table 1. In the total population, there was a baseline prevalence of 4.1% and 9.1% for sarcopenia and impaired exocrine pancreatic function, respectively. Both conditions were more common in the older age group.

**TABLE 1:** Baseline demographic and clinical participant characteristics.

Parameter	Total (*n* = 5,598)			Age < 65 yrs. (*n* = 4,123)			Age ≥ 65 yrs. (*n* = 1,475)		
	No sarcopenia (*n* = 5,368)	Sarcopenia (*n* = 230)	*p*	No sarcopenia (*n* = 4,045)	Sarcopenia (*n* = 78)	*p*	No sarcopenia (*n* = 1,323)	Sarcopenia (*n* = 152)	*p*
Age, yrs.	53.0 (22.0)	71.0 (18.0)	**<0.001**	48.0 (17.0)	53.0 (12.0)	**0.001**	70.0 (8.0)	74.0 (9.0)	**<0.001**
Sex, *n* (%)			0.377			0.306			0.731
Male	2,571 (47.9)	117 (50.9)		1,890 (46.7)	41 (52.6)		681 (51.5)	76 (50)	
Female	2,797 (52.1)	113 (49.1)		2,155 (53.3)	37 (47.4)		642 (48.5)	76 (50)	
Smoking status, *n* (%)			0.089			0.692			0.324
Never	1,983 (36.9)	93 (40.4)		1,363 (33.7)	26 (33.3)		620 (46.9)	67 (44.1)	
Former	2,064 (38.5)	95 (41.3)		1,449 (35.8)	25 (32.1)		615 (46.5)	70 (46.1)	
Current	1,321 (24.6)	42 (18.3)		1,233 (30.5)	27 (34.6)		88 (6.7)	15 (9.9)	
Body mass index, kg/m^2^	27.69 (6.40)	24.51 (4.19)	**<0.001**	27.09 (6.59)	23.3 (3.98)	**<0.001**	29.11 (5.54)	24.89 (4.23)	**<0.001**
Diabetes mellitus, *n* (%)	598 (11.1)	29 (12.6)	0.489	294 (7.3)	9 (11.5)	0.152	304 (23.0)	20 (13.2)	**0.006**
Physical inactivity^a^, *n* (%)	2,702 (50.3)	112 (48.7)	0.667	1,909 (47.2)	34 (43.6)	0.576	793 (59.9)	78 (51.3)	0.143
Handgrip strength, kg	35.0 (19.5)	22.5 (15.5)	**<0.001**	36.5 (19.5)	25.3 (17.0)	**<0.001**	31.5 (17.5)	22.5 (13.9)	**<0.001**
Skeletal muscle mass index, kg/m^2^	9.11 (2.90)	7.46 (2.65)	**<0.001**	9.11 (2.94)	7.62 (2.72)	**<0.001**	9.12 (2.80)	6.93 (2.63)	**<0.001**
Muscle specific strength^b^, kg/kg	1.40 (0.34)	1.26 (0.23)	**<0.001**	1.43 (0.32)	1.25 (0.26)	**<0.001**	1.31 (0.37)	1.27 (0.22)	**0.007**
Fecal elastase, μg/g	460 (248)	455 (245)	0.357	473 (243)	460 (280)	0.456	423 (243)	455 (235)	0.167
Impaired pancreatic exocrine function^c^, *n* (%)	485 (9.0)	27 (11.7)	0.164	323 (8.0)	14 (17.9)	**0.001**	162 (12.2)	13 (8.6)	0.182

All data are presented as median (IQR) unless indicated otherwise. Bold values denote statistical significance at the *p* < 0.05 level. Significant differences between subjects with and without sarcopenia were tested using independent *t*-test and Chi-squared test for continuous and categorical variables, respectively.

^a^Less than 1 h of exercise per week.

^b^Handgrip strength per kg of skeletal muscle mass.

^c^Fecal elastase ≤ 200 μg/g.

### 3.2 Cross-sectional association between exocrine pancreatic function and sarcopenia

**FIGURE 2:**
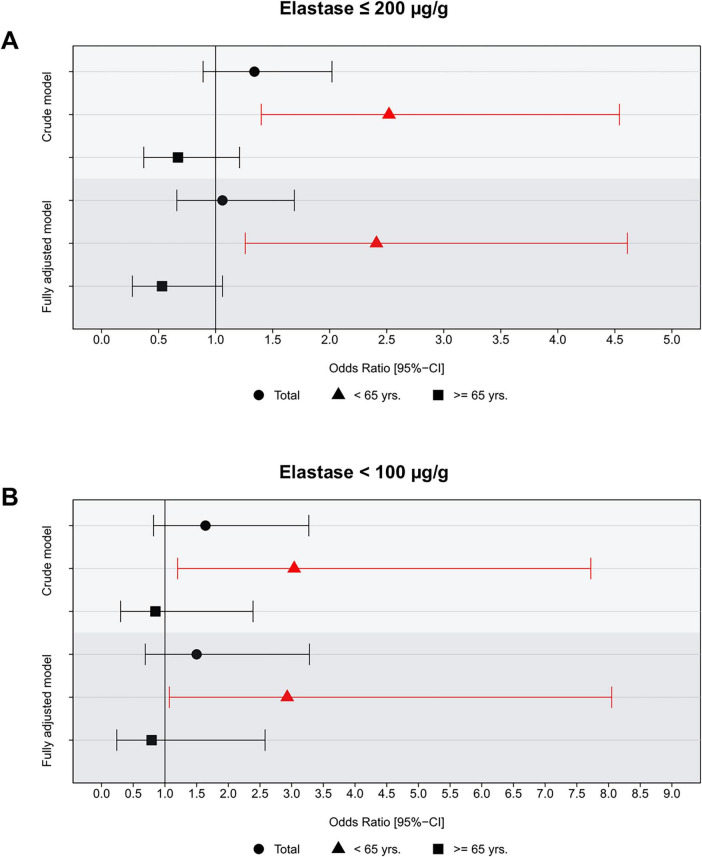
Association of impaired **(A)** and severely impaired exocrine pancreatic function **(B)** with sarcopenia stratified by age and in the total population based on logistic regression analysis. The fully adjusted model includes age, sex, BMI, smoking status, diabetes mellitus, and physical inactivity as covariates.

**FIGURE 3:**
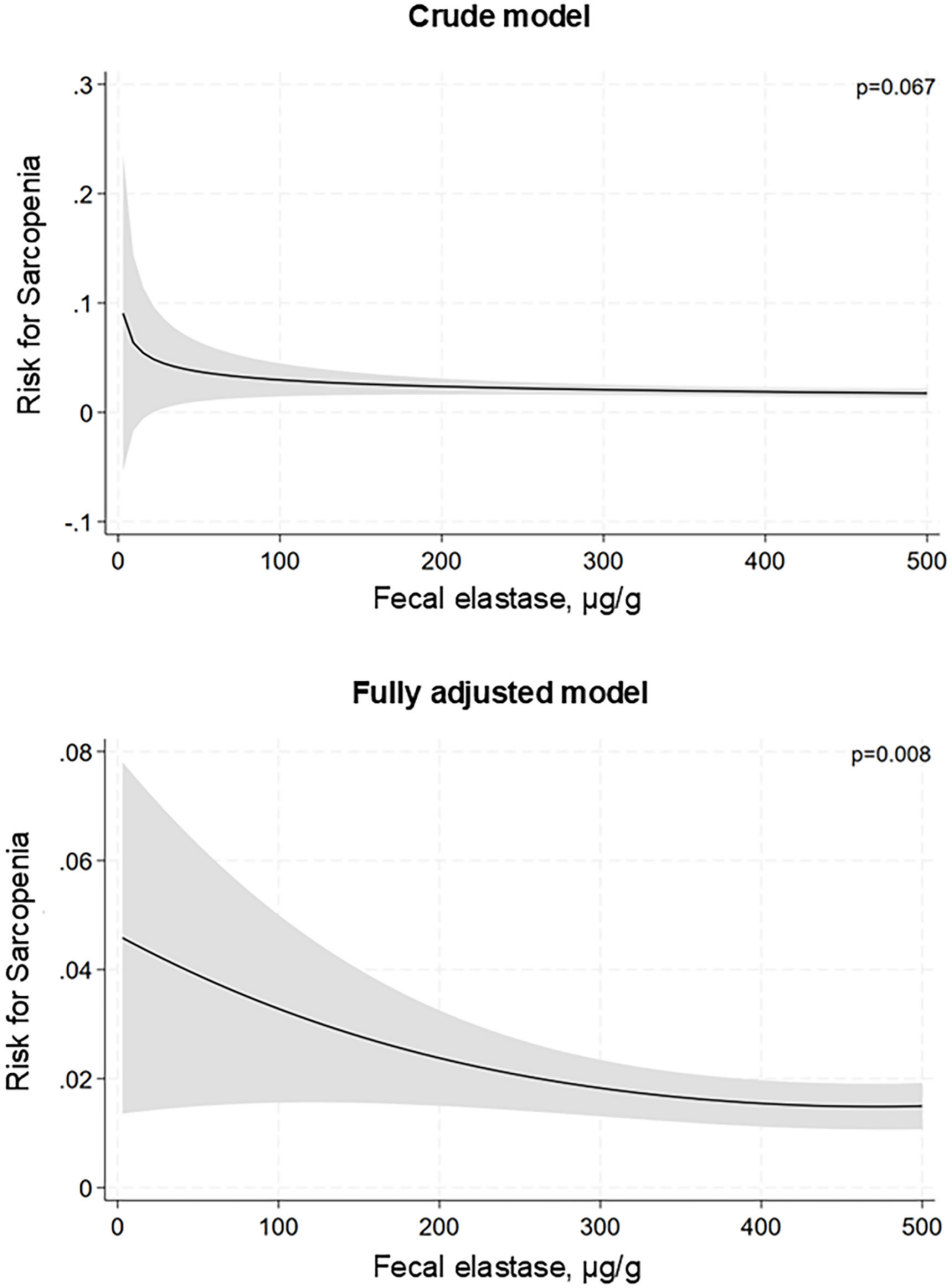
Association of fecal elastase concentration with sarcopenia in participants younger than 65 years. Data is presented as risk difference based on logistic regression analysis. The fully adjusted model includes age, sex, BMI, smoking status, diabetes mellitus, and physical inactivity as covariates.

The results of cross-sectional logistic regression analyses are summarized in Figures 2, 3. In the total population, univariable analysis showed no significant association between reduced exocrine pancreatic function and sarcopenia (*p* = 0.165). However, when data were stratified by age group, we found no association in persons 65 years of age or older (*p* = 0.185), but there was a higher prevalence of sarcopenia in individuals younger than 65 years of age (OR [95% CI]: 2.52 [1.40; 4.54]). These results remained robust in the multivariable models. In the fully adjusted model, we observed a greater prevalence of sarcopenia (OR [95% CI]: 2.41 [1.26; 4.61]) in the younger subjects while there was a tendency toward an inverse association in older study participants (OR [95% CI]: 0.53 [0.27; 1.06]) (Figure 2A). Associations with severe exocrine pancreatic impairment were congruent in direction and greater in strength (Figure 2B). When we performed additional analyses including fecal elastase levels as a continuous independent variable in the logistic regression models, there were no significant associations in neither the total population nor the older subjects. Yet, in the younger participants we found a significant non-linear association between fecal elastase level and risk of sarcopenia when adjusted for confounders (Figure 3).

### 3.3 Cross-sectional association between exocrine pancreatic function and muscle parameters

When testing the relation of impaired exocrine pancreatic function with individual muscle parameters, we found positive associations with muscle mass and strength but an inverse one with muscle specific strength (Table 2). However, significance of these associations essentially was limited to the univariable model as there was no significant relation with any of the three muscle parameters in the fully adjusted models.

**TABLE 2:** Association of impaired exocrine pancreatic function and muscle parameters stratified by age and in the total population.

Parameter	Total		<65 years		≥65 years	
	(*n* = 5,598)	*p*	(*n* = 4,123)	*p*	(*n* = 1,475)	*p*
Skeletal muscle mass index, kg/m2						
Crude model	0.561 (0.403; 0.719)	**<0.001**	0.726 (0.532; 0.920)	**<0.001**	0.305 (0.032; 0.578)	**0.029**
Fully adjusted model	0.060 (−0.006; 0.126)	0.073	0.056 (−0.024; 0.135)	0.170	0.073 (−0.044; 0.189)	0.221
Muscle Strength, kg						
Crude model	1.514 (0.417; 2.611)	**0.007**	2.970 (1.625; 4.315)	**<0.001**	0.179 (−1.535; 1.892)	0.838
Fully adjusted model	0.34 (−0.586; 0.654)	0.914	−0.094 (−0.870; 0.682)	0.813	−0.047 (−1.015; 0.921)	0.924
Muscle specific strength, kg/kg						
Crude model	−0.038 (−0.061; −0.014)	**0.002**	−0.022 (−0.049; 0.006)	0.128	−0.040 (−0.083; 0.004)	0.072
Fully adjusted model	−0.008 (−0.030; 0.014)	0.493	−0.007 (−0.034; 0.019)	0.583	−0.013 (−0.053; 0.026)	0.508

Data are presented as univariable and multivariable adjusted β coefficients and 95% confidence interval. Full model adjusted for age, sex, BMI, smoking status, diabetes mellitus, and physical inactivity. Bold values denote statistical significance at the *p* < 0.05 level.

### 3.4 Longitudinal association between exocrine pancreatic function and sarcopenia

**FIGURE 4:**
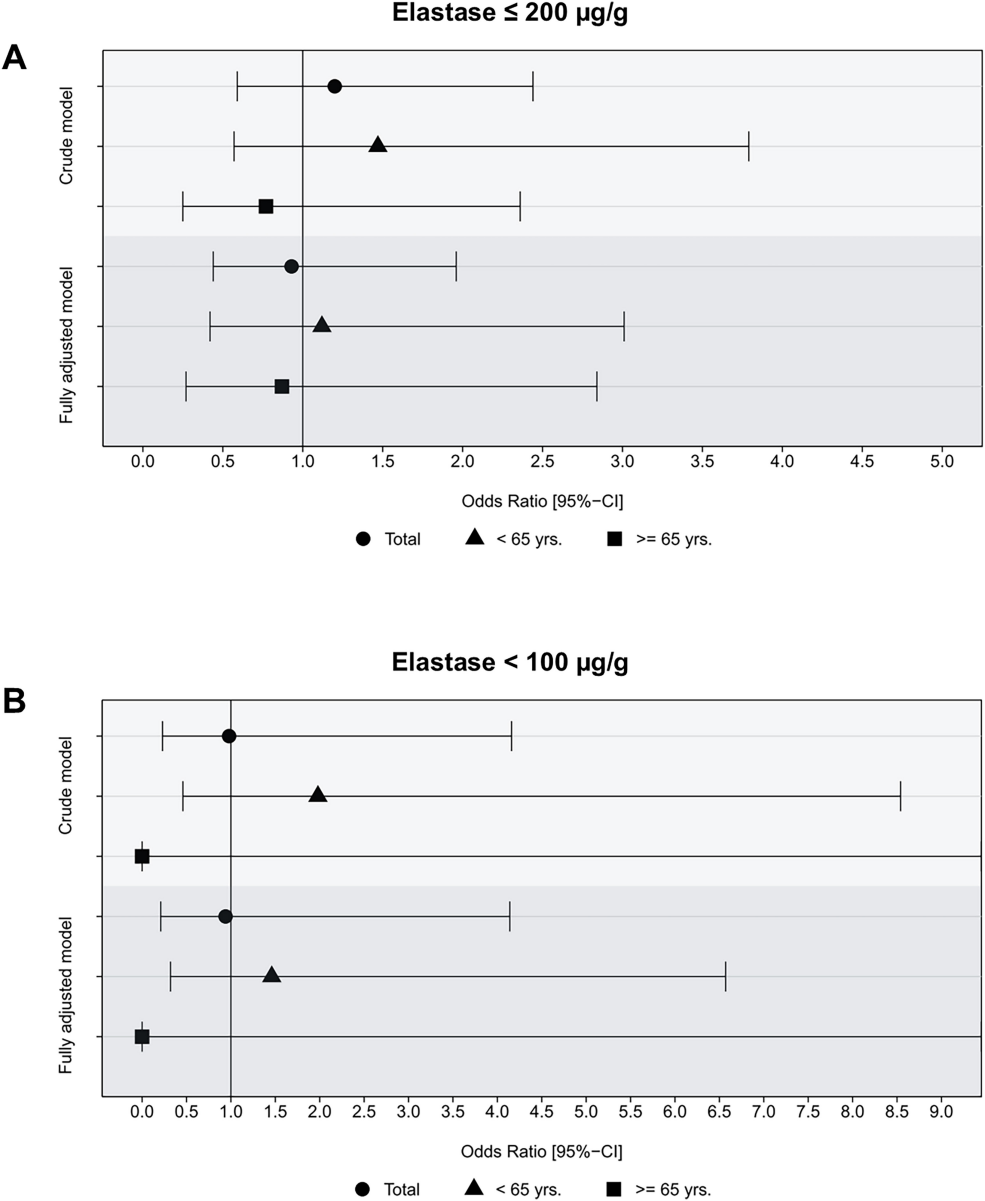
Association of impaired **(A)** and severely impaired exocrine pancreatic function **(B)** with incident sarcopenia stratified by age and in the total population based on logistic regression analysis. The fully adjusted model includes age, sex, BMI, smoking status, diabetes mellitus, and physical inactivity as covariates.

**FIGURE 5:**
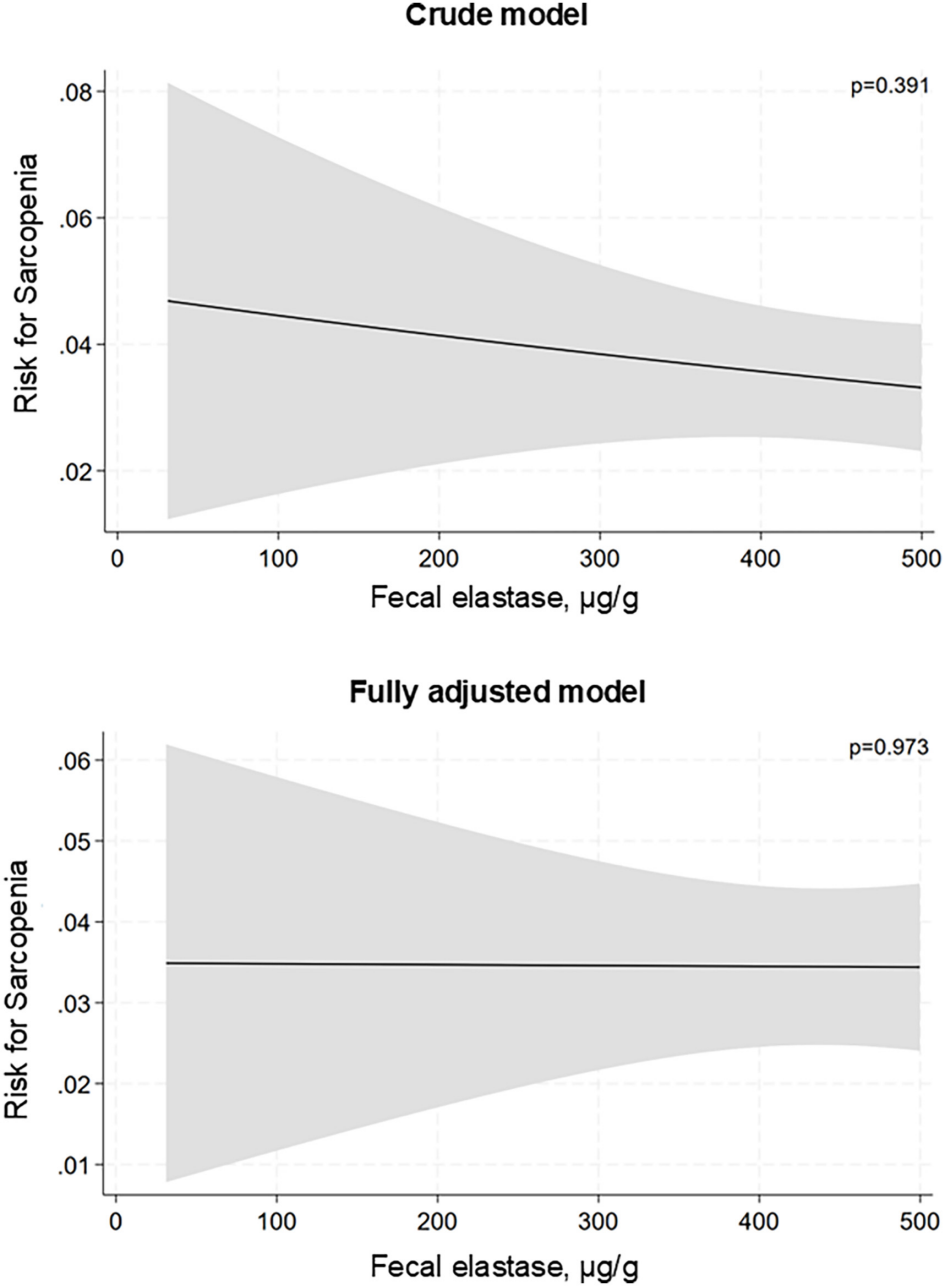
Association of fecal elastase concentration with incident sarcopenia in participants younger than 65 years. Data is presented as risk difference based on logistic regression analysis. The fully adjusted model includes age, sex, BMI, smoking status, diabetes mellitus, and physical inactivity as covariates.

During A total of 1,587 individuals participating in the follow-up examinations of SHIP-TREND-1 had complete data on muscle assessment available. Among these participants, there were 95 cases (6.0%) of new onset sarcopenia. Development of sarcopenia was more frequently seen in subjects with a baseline age of 65 years or older than in those being younger (19.4% vs. 3.4%). There was no significant association between reduced exocrine pancreatic function and new onset sarcopenia, neither in univariate nor in multivariate logistic regression (Figure 4A). This absence of an association was seen in both the total population as well as age stratified analysis. There was also no association when we analyzed only subjects with severely impaired exocrine function (Figure 4B). Notably, no participant with severe exocrine pancreatic impairment 65 years or older developed sarcopenia during the follow-up. Accordingly, we did not observe any significant associations between fecal elastase concentration and development of sarcopenia, when considered as a continuous variable. A null association was not only found for the total and older population but also for the younger participants (Figure 5).

### 3.5 Longitudinal association between exocrine pancreatic function and muscle parameters

Regarding the associations between impaired exocrine pancreatic function at baseline and changes in muscle parameters during follow-up, we observed discrepant findings (Table 3). While we found no relation with both muscle strength and quality, impaired exocrine pancreatic function was significantly linked to a decline in skeletal muscle mass. This association was exclusively seen in the younger, but not the older, participants and remained basically unchanged in the fully adjusted model.

**TABLE 3:** Association of impaired exocrine pancreatic function and changes in muscle parameters during follow-up stratified by age and in the total population.

Parameter	Total		<65 years		≥65 years	
	(*n* = 1,587)	*p*	(*n* = 1,335)	*p*	(*n* = 252)	*p*
Skeletal muscle mass index, kg/m2						
Crude model	−0.185 (−0.327; −0.043)	**0.011**	−0.234 (−0.390; −0.079)	**0.003**	0.036 (−0.313; 0.385)	0.839
Fully adjusted model	−0.135 (−0.275; 0.005)	0.059	−0.187 (−0.341; −0.033)	**0.018**	0.052 (−0.295; 0.399)	0.767
Muscle strength, kg						
Crude model	−0.610 (−1.654; 0.433)	0.251	−0.957 (−2.132; 0.217)	0.110	1.296 (−0.788; 3.379)	0.222
Fully adjusted model	0.137 (−0.876; 1.151)	0.790	−0.259 (−1.415; 0.896)	0.660	1.751 (−0.301; 3.803)	0.094
Muscle specific strength, kg/kg						
Crude model	0.006 (−0.041; 0.052)	0.809	0.000 (−0.051; 0.052)	0.985	0.043 (−0.061; 0.146)	0.420
Fully adjusted model	0.028 (−0.019; 0.075)	0.239	0.021 (−0.031; 0.074)	0.421	0.056 (−0.050; 0.161)	0.299

Data are presented as univariable and multivariable adjusted β coefficients and 95% confidence interval. Full model adjusted for age, sex, BMI, smoking status, diabetes mellitus, and physical inactivity. Bold values denote statistical significance at the *p* < 0.05 level.

## 4 Discussion

Here, we demonstrate that in an asymptomatic general population without history of pancreatic diseases impaired exocrine pancreatic function is associated with sarcopenia. Moreover, our data indicate that subclinical exocrine pancreatic impairment could increase the risk of sarcopenia in younger subjects by accelerating muscle mass decline.

Previous studies have linked exocrine pancreatic insufficiency to sarcopenia in patients with chronic pancreatitis or pancreatic cancer (22–25). However, in these disorders other factors, such as inflammation and pain-related reduced food intake, can expedite the loss of muscle mass and function as well. Thus, nutritional management, including enzyme replacement therapy in case of exocrine pancreatic insufficiency, is generally indicated in these patients. By contrast, outside a clinical context asymptomatic exocrine pancreatic impairment will remain undiagnosed and untreated. Until now, subclinical exocrine pancreatic impairment, despite being a fairly common condition, has not been linked to any negative health outcome that could justify population-wide screening or initiation of pancreatic enzyme replacement therapy. Our study provides a novel perspective since asymptomatic study participants showed reduced exocrine pancreatic function which may have fostered the development of sarcopenia. The fact that we observed this relation exclusively in younger subjects and even after adjustment for several confounders, suggests impaired exocrine pancreatic function as an independent risk factor of secondary sarcopenia. While the finding of an association only in younger adults is intriguing, there are two potential explanations why we might not observe an association in older participants. First, one must consider that this result is due to survival bias. Second, it is also possible that there were other competing causes of sarcopenia in older subjects, such as age or chronic disease. As our study cannot definitely answer why reduced exocrine pancreatic function was only linked to sarcopenia in younger persons, subsequent investigations are clearly warranted.

Likewise, the discrepancy between cross-sectional and longitudinal analyses deserves more detailed consideration. As these inconsistent results could stem from reverse causality or residual confounding, our findings should be interpreted with caution. Observing only a significant association with muscle mass decline but not incident sarcopenia during following-up also seems to weaken our hypothesis. However, this might merely result from the limited follow-up duration or lowered statistical power of longitudinal analyses. Consequently, for now it seems inappropriate to derive any strong recommendations regarding treatment of subjects with asymptomatic impaired exocrine function without confirmative research.

Finally, our results raise the question of the biological mechanism how subclinical exocrine pancreatic insufficiency could lead to sarcopenia. Subjects with reduced exocrine pancreatic function have a higher body weight and the association with sarcopenia persisted even after adjustment for BMI. Thus, weight loss due to malabsorption of energy-providing macronutrients is not a plausible mechanistic explanation. Nevertheless, reduced exocrine pancreatic function could also result in the malassimilation of micronutrients that are critical to muscle health. In particular, lower levels of vitamin D (26–28) and zinc (29, 30) have repeatedly been linked to sarcopenia and deficiency in both nutrients is associated with manifest exocrine pancreatic insufficiency (31, 32). However, causal contributions to the development of sarcopenia have yet to be confirmed. Further, it has to be remembered that in Germany, as in most other countries, vitamin D status is largely influenced by endogenous synthesis via sun exposure (33). Also, there are adaptive mechanisms in zinc absorption to ensure its homeostasis (34).

Another potentially mediating factor between impaired exocrine pancreatic function and sarcopenia could be the intestinal microbiome. In a previous study (35), exocrine pancreatic function has been identified as the most important host factor shaping the human gut microbiota. Furthermore, in this investigation reduced exocrine pancreatic function was associated with a pro-inflammatory enterotype characterized by higher *Prevotella* and lower *Bacteroides* abundances. Similar changes in intestinal microbiota composition have been reported in the context of sarcopenia (36). For instance, a significant increase in *Bacteroides* was found in elderly women participating in a 12-weeks aerobic exercise intervention (37) while another study showed a lower *Bacteroides/Prevotella* ratio in elderly persons with frailty (38).

Elucidating the underlying mechanisms in the relation between reduced exocrine pancreatic function and sarcopenia is beyond the scope of this work. Because there are multiple plausible and not mutually exclusive mediating factors, additional research on the mechanisms that could cause an increased risk of sarcopenia in people with exocrine pancreatic impairment is highly warranted.

Despite large population size, cross-sectional and longitudinal analyses with meticulous examination of exposure and outcome, our work has some limitations that need to be acknowledged. As this was an observational study, we cannot rule out residual confounding through unknown or unmeasured factors. Yet, we controlled for relevant confounders and the results remained mainly consistent across crude and adjusted models, which limits this possibility. Moreover, our conclusions regarding a causal relation remain confined as we did not observe significant associations with incident sarcopenia. Nevertheless, despite the relatively short follow-up duration of 5 years we found reduced exocrine pancreatic function already to be associated with muscle mass decline, which supports the clinical relevance of exocrine pancreatic impairment in this context. As our analyses were performed in a distinct population of Northeast Germany, our findings however may not necessarily be generalized to other populations of different ethnic or socio-demographic background and require confirmation. Further, selection bias inherent to population-based studies should be considered. Last, there is a chance of classification bias as we employed self-reported medical data to rule out any underlying pancreatic disease. Also, fecal elastase testing is known to have limited sensitivity in mild EPI (12, 13). However, the observed prevalence of subclinical reduced exocrine pancreatic function in our cohort is consistent with previously reported data in the general population (14). Thus, it is unlikely that participants were systematically misclassified as healthy while actually suffering from pancreatic diseases. In addition, although we followed current international recommendations on diagnosis of sarcopenia, it must be acknowledged that there is still no international consensus on its operational definition, which could limit comparability of our findings. While choice of methods and thresholds clearly will have an impact on sarcopenia prevalence, our diagnostic approach seems supported by existing literature on the epidemiology of sarcopenia (39). Thus, the risk of misclassification in terms of outcome appears low. Still results should be interpreted with caution in terms of clinical implications.

In conclusion, we show that impaired exocrine pancreatic function could be linked with sarcopenia also in healthy individuals without any known history of pancreatic disease, especially those of younger age. While our work requires confirmation, our findings of a potentially harmful effect on muscle health suggest that in persons with reduced fecal elastase levels despite being asymptomatic careful monitoring of muscle health could be indicated. However, before any definite implications for clinical practice, such as early initiation of pancreatic enzyme replacement or nutrition therapy, can be derived, according clinical trials are clearly warranted.

## Data Availability

The datasets presented in this article are not readily available because restrictions apply to the availability of data from the “Study of Health of Pomerania.” Data are however available upon reasonable request at https://transfer.ship-med.uni-greifswald.de/FAIRequest/?lang=en and with permission of the University Medicine Greifswald. Requests to access the datasets should be directed to AA, ali.aghdassi@med.uni-greifswald.de.
